# Senescent synovial fibroblasts accumulate prematurely in rheumatoid arthritis tissues and display an enhanced inflammatory phenotype

**DOI:** 10.1186/s12979-019-0169-4

**Published:** 2019-11-05

**Authors:** Manuel J. Del Rey, Álvaro Valín, Alicia Usategui, Sandra Ergueta, Eduardo Martín, Cristina Municio, Juan D. Cañete, Francisco J. Blanco, Gabriel Criado, José L. Pablos

**Affiliations:** 1 Grupo de Enfermedades Inflamatorias y Autoinmunes, Instituto de Investigación Hospital 12 de Octubre (i+12), Madrid, Spain; 2 Unitat d’Artritis, Servei de Reumatologia, Hospital Clínic de Barcelona and Institut d’Investigacions Biomèdiques August Pí i Sunyer, Barcelona, Spain; 3 Laboratorio de Investigación Osteoarticular y del Envejecimiento, Instituto de Investigación Biomédica de A Coruña, INIBIC, A Coruña, Spain; 4 Centro de Investigación, Hospital 12 de Octubre, 28041 Madrid, Spain; 5 Servicio de Reumatología, Hospital 12 de Octubre, Universidad Complutense de Madrid, 28041 Madrid, Spain

**Keywords:** Rheumatoid arthritis, Synovial fibroblasts, Aging, Cell senescence, SASP

## Abstract

**Background:**

Accumulation of senescent cells has been associated with pro-inflammatory effects with deleterious consequences in different human diseases. The purpose of this study was to analyze cell senescence in human synovial tissues (ST), and its impact on the pro-inflammatory function of synovial fibroblasts (SF).

**Results:**

The expression of the senescence marker p16INK4a (p16) was analyzed by immunohistochemistry in rheumatoid arthritis (RA), osteoarthritis (OA), and normal ST from variably aged donors. The proportion of p16(+) senescent cells in normal ST from older donors was higher than from younger ones. Although older RA and OA ST showed proportions of senescent cells similar to older normal ST, senescence was increased in younger RA ST compared to age-matched normal ST. The percentage of senescent SA-β-gal(+) SF after 14 days in culture positively correlated with donor’s age. Initial exposure to H_2_O_2_ or TNFα enhanced SF senescence and increased mRNA expression of *IL6*, *CXCL8*, *CCL2* and *MMP3* and proteins secretion. Senescent SF show a heightened *IL6*, *CXCL8* and *MMP3* mRNA and IL-6 and IL-8 protein expression response upon further challenge with TNFα. Treatment of senescent SF with the senolytic drug fenofibrate normalized *IL6*, *CXCL8* and *CCL2* mRNA expression.

**Conclusions:**

Accumulation of senescent cells in ST increases in normal aging and prematurely in RA patients. Senescence of cultured SF is accelerated upon exposure to TNFα or oxidative stress and may contribute to the pathogenesis of synovitis by increasing the production of pro-inflammatory mediators.

## Background

A common factor to aging associated diseases is a chronic, low-grade inflammation, that has been termed “inflammaging” [1, 2]. The cellular basis of this process consists of a cellular pro-inflammatory phenotype, referred to as “senescence-associated secretory phenotype” (SASP), characterized by enhanced production of cytokines, chemokines and matrix metalloproteinases that has been linked to cell senescence [3–5]. Senescent cells, characterized by the expression of p16INK4a and irreversible cell-cycle arrest, accumulate in tissues during normal aging, and are thought to contribute to organ dysfunction in different age-associated diseases [6–10]. This concept has been explored in osteoarthritis (OA), a joint disease tightly linked to aging. Increased senescent chondrocytes with a pro-inflammatory SASP phenotype have been demonstrated in OA cartilage and their transplant to normal joints induce joint damage similar to OA [11–14]. Removal of senescent cells by transgenic technology in an experimental OA model has also demonstrated their deleterious effects, providing the basis for senolysis as a potential therapy in OA [15].

The relationship between aging and arthritis is less clear in other chronic inflammatory joint conditions such as rheumatoid arthritis (RA). Epidemiology of RA shows that its incidence increases with aging, with the sharpest incidence rise between the fourth and sixth decades of life [16]. Aging of the T-cell compartment of the immune system may favor autoimmunity, and this mechanism has been proposed to explain the relationship between aging and RA [17, 18]. However, the potential participation of aging-associated cell changes in the local synovial environment has not been studied. Chronic inflammatory diseases are characterized by increased oxidative stress and ROS production, one of the most relevant contributors to the genomic and mitochondrial damage that characterizes the cellular aging process. It is therefore expected that local cell aging be present in the inflamed synovium. Tissue inflammation and cell senescence have been shown to synergize in the production of pro-inflammatory mediators such as IL-6 that, in turn, activates cellular reprogramming [19]. This reprogramming is interpreted as an attempt to repair tissue damage, but proof of this plasticity in synovitis or other human inflammatory tissues is lacking.

The main resident cells in RA synovial tissue (ST) are synovial fibroblasts (SF) which contribute to the pathogenesis of arthritis by synthesizing a variety of mediators that are very similar to those associated to the SASP, notably IL-6, MMPs and chemokines [20]. Fibroblast senescence has been observed in other normal aged tissues and in pathological tissues such as cancer or fibrosis [21, 22]. In normal skin, fibroblast senescence has been linked to aging and to the acquisition of a matrix degradation and pro-inflammatory phenotype [8, 23]. Here, we describe the accumulation of senescent cells in normal aged and arthritic ST and characterize the phenotype of senescent SF.

## Results

### p16 and NANOG expression in ST

**Fig. 1:**
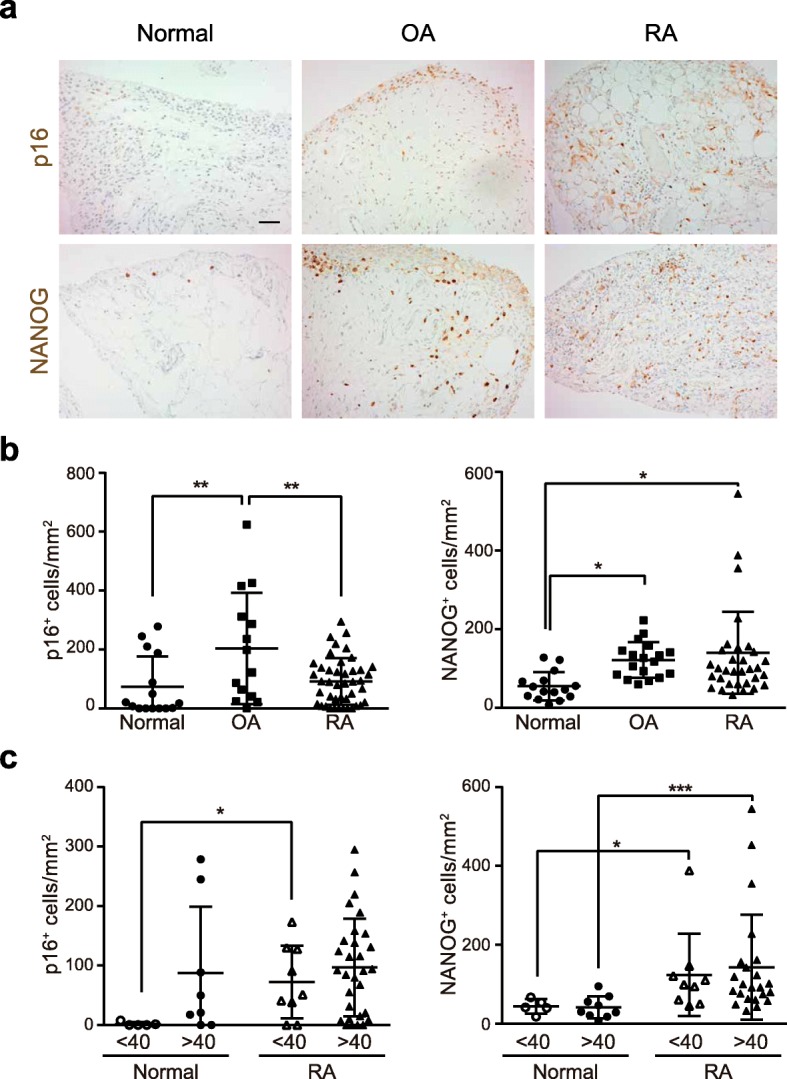
Expression of p16 and NANOG in ST. **a** IHC detection of p16 and NANOG in normal, OA and RA ST (Bar 50 μm). **b** Density of p16(+) and NANOG(+) cells in the different groups (*ANOVA). **c** Density of p16(+) and NANOG(+) cells in normal and RA ST stratified by age: under (< 40) and over (> 40) 40 years old (*Mann-Whitney)

Nuclear expression of the p16 senescent marker was detected by immunohistochemistry (IHC) in all OA, RA and normal ST groups (Fig. 1a). The proportion of biopsies showing presence of p16(+) cells was higher in RA (37/43, 86%) and OA (13/14, 92.9%) than in normal groups (10/15, 66.7%). Consistent with the older age of OA patients (69.9 ± 5.7 vs 40 ± 16.06 in control group and 54.4 ± 14.3 in RA group), OA synovia showed a significantly higher density of p16(+) cells (Fig. 1b). Since the mean and median age of the healthy donor population were 40 and 44 years old, respectively, we set up a cut-off age of 40 years to dissect the contribution of age to the abundance of p16 and NANOG. Expression of p16 in normal ST was higher in old (age > 40 years old) compared with young (age < 40 years old) donors. Younger RA patients showed a significant increase of p16(+) cell density compared with age-matched healthy donors, whereas older RA patients showed levels similar to the normal controls group (Fig. 1c). Scattered senescent p16(+) cells were observed both through lining and sublining areas in all normal and OA/RA groups. Double immunofluorescent (IF) labelling confirmed that most p16(+) nuclei colocalized to hsp47(+) SF [24] or CD68(+) macrophages (see in Additional file 1: Figure S1).

Expression of the reprogramming marker NANOG, previously shown associated to cell senescence [19], was significantly increased in OA and RA groups compared to normal ST (Fig. 1a and b). No age-related differences in NANOG expression were observed in either normal or in RA ST groups (Fig. 1c).

### Cell senescence in cultured SF

**Fig. 2:**
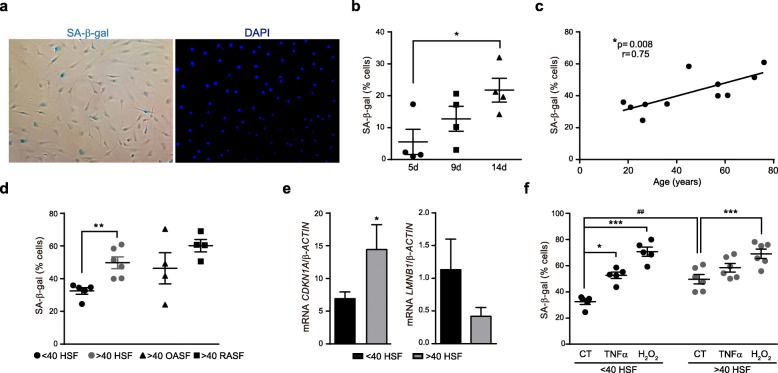
Analysis of senescence in 14-day SF cultures. **a** SA-β-gal activity and DAPI staining. **b** Time-dependent expansion of SA-β-gal(+) in HSF cultures (*n* = 4) (*ANOVA). **c** Correlation between the frequency of SA-β-gal(+) SF and donor’s age (*n* = 11). **d** Frequency of SA-β-gal(+) from < 40 (*n* = 5) and > 40 (*n* = 6) HSF, and from > 40 OASF (*n* = 4) and > 40 RASF (*n* = 4) cultures (*Mann-Whitney). **e** *CDKN1A* and *LMNB1* mRNA expression in < 40 (*n* = 3) and > 40 (*n* = 5) HSF cultures (*Mann-Whitney). **f** Frequency of SA-β-gal(+) from < 40 (*n* = 5) and > 40 (*n* = 6) HSF cultures treated with TNFα, H_2_O_2_ or untreated (CT) (*Friedman, ^##^Mann-Whitney)

Replicative senescence was induced in SF from healthy donors after 14-day culture. Cultured SF usually reached confluence at day 8 after seeding and at day 14 most SF were arrested in G_0_/G_1_ phase but significant cell death was not observed (data not shown). To determine the proportion of senescent cells under these conditions in the different groups we detected lysosomal β-galactosidase activity [25, 26] (Fig. 2a). Frequency of SA-β-gal(+) senescent cells in SF cultures increased proportionally to the number of days in culture and was significantly higher at day 14 than at day 5 of culture (Fig. 2b). Under these conditions, we determined the proportion of senescent cells in SF cultures obtained from differently aged healthy donors. The percentage of SA-β-gal(+) cells in 14-day cultures positively correlated with age (r = 0.75/*p* = 0.008) and was significantly higher in old (> 40 years old) than in young healthy SF (Fig. 2c and d). SF cultures from old arthritic patients (OA or RA) showed a proportion of SA-β-gal(+) that was similar to the similarly aged (> 40 years old) group of healthy donors. Increased senescence of old normal SF compared to younger counterparts was confirmed by the mRNA expression of additional markers of senescence, i. e., enhanced *CDKN1A* and reduced *LMNB1* [27, 28] (Fig. 2e).

To evaluate the influence of oxidative or inflammatory stress on SF senescence we developed a model of stress-induced senescence. To this aim, 14-day cultures of normal SF were briefly exposed to TNFα or H_2_O_2_ before the onset of senescence (day 2 for TNFα, day 5 for H_2_O_2_) and the presence of SA-β-gal(+) senescent cells was analyzed at the end of the experiment. Both TNFα and H_2_O_2_ significantly increased the proportion of senescent SA-β-gal(+) in young SF. Analysis of old SF confirmed an elevated proportion of senescent cells in basal conditions and showed that TNFα and H_2_O_2_ induced a more limited increase, only significant upon H_2_O_2_ exposure (Fig. 2f).

Collectively, these results show that SF cultures from younger individuals are highly susceptible to stress-induced senescence, whereas SF from older individuals show increased spontaneous senescence in 14-day cultures.

### Expression of SASP mediators by senescent SF

Next, we used the model of SF stress-induced senescence as described above to analyze whether SF senescence is also paralleled by increased expression of the pro-inflammatory factors characteristic of the SASP.

**Fig. 3:**
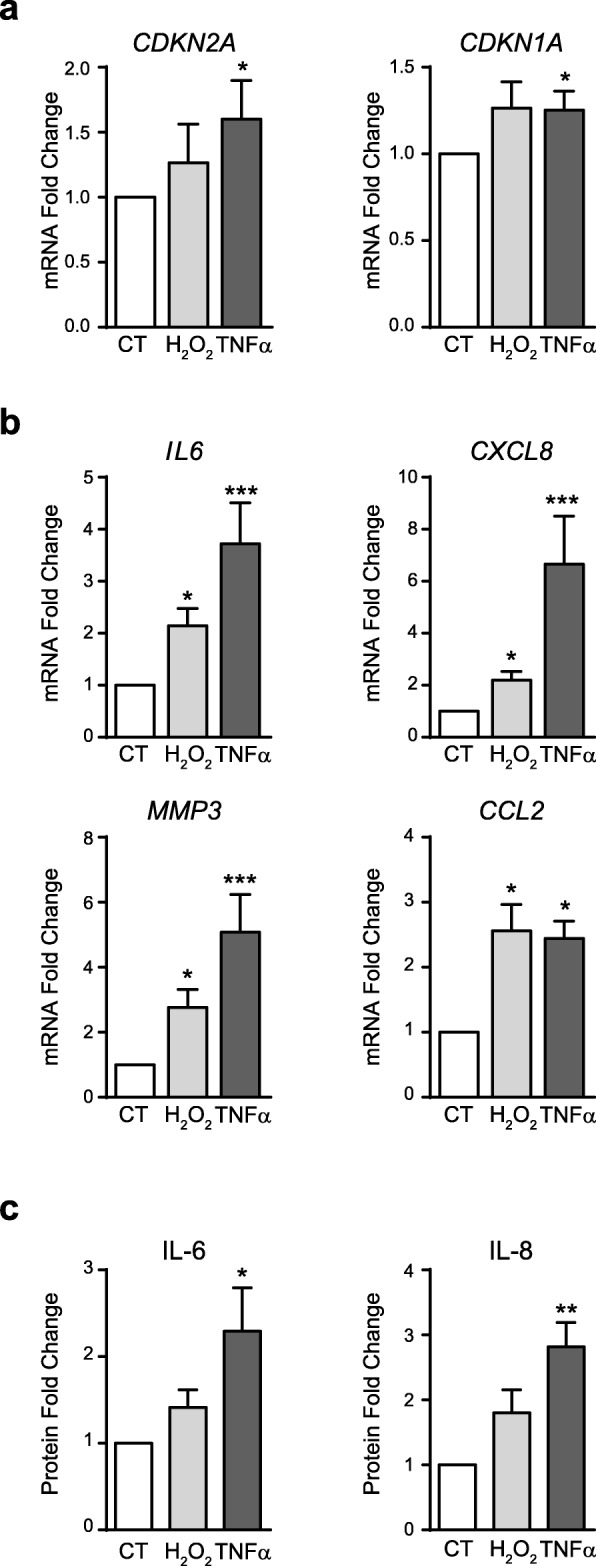
Analysis of senescent markers and SASP mediators in stress-induced senescent SF. HSF in 14-day cultures subjected to stress-induced senescence with H_2_O_2_ or TNFα. **a** Change in *CDKN2A* and *CDKN1A* mRNA expression (*n* = 8). **b** Change in *IL6*, *CXCL8*, *MMP3* and *CCL2* mRNA expression (*n* = 12). **c** Change in IL-6 and IL-8 protein levels (*n* = 8). (*Wilcoxon test)

We collected RNA and supernatants from control or senescent SF induced by H_2_O_2_ or TNFα in 14-day cultures. mRNA expression of the p16-encoding gene, *CDKN2A*, and *CDKN1A* confirmed increased SF senescence (Fig. 3a), and mRNA expression of pro-inflammatory SASP-associated factors: *IL6* and *CXCL8*, monocyte recruitment chemokine *CCL2* and matrix metallopeptidase protein *MMP3* were determined. All these factors were up-regulated by TNFα- and, more variably, by H_2_O_2_-induced senescence (Fig. 3b). These findings were mirrored by a similar increase in the levels of secreted IL-6 and IL-8 proteins in culture supernatants, also more consistently with TNFα (Fig. 3c).

Since the pro-inflammatory cytokine TNFα is a strong short-term inducer of these factors in SF, we examined the kinetics of cytokine expression upon TNFα treatment to confirm that the observed late up-regulation of inflammatory gene expression is associated to senescence rather than to a persistent transcriptional response to TNFα. Kinetics analysis indicated that expression of *IL6* and *CXCL8* was comparable between control and TNFα senescent SF after 8 days in culture, started to increase in TNFα-senescent cultures by day 11 in culture and reached the peak expression by day 14, the endpoint of senescent cultures. These findings rule out a direct contribution of the early TNFα challenge to the late SASP expression (see in Additional file 2: Figure S2).

**Fig. 4:**
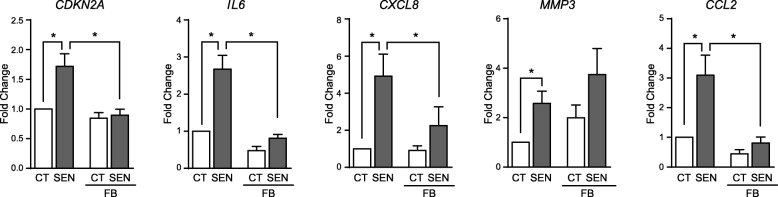
Effect of fenofibrate treatment in TNFα-induced senescent SF. 14-day senescent (SEN) and control (CT) SF were treated with fenofibrate (FB, 25 μM) for 72 h. Graphics show the changes in *CDKN2A* and SASP factors *IL6*, *CXCL8*, *MMP3* and *CCL2* mRNA expression in relation to untreated CT SF (*n* = 7) (*Wilcoxon test)

These results indicate that stress-induced senescence enhanced the expression of factors characteristic of the SASP in SF, and that the up-regulation of the inflammatory genes is temporally associated to the acquisition of senescence rather than to persistent transcriptional effects. Under these circumstances, pharmacological targeting of senescent cells can provide a therapeutic opportunity to reduce senescence-associated inflammation. To test this hypothesis, we treated TNFα-induced senescent SF for 72 h with fenofibrate, a PPARα agonist recently been reported to have potent senolytic and senomorphic activity in senescent chondrocytes and tumour cell lines [29, 30]. Fenofibrate treatment of TNFα-senescent SF provoked a reduction of *CDKN2A* expression to levels comparable of control SF (Fig. 4). Fenofibrate did not induce increased cell death as assessed by microscopy or lactate dehydrogenase (LDH) activity in supernatants, thus pointing to a senomorphic rather that senolytic effect. This reduction in *CDKN2A* expression was accompanied by a significant decrease in the expression of *IL6*, *CXCL8* and *CCL2* but not that of *MMP3* (Fig. 4).

**Fig. 5:**
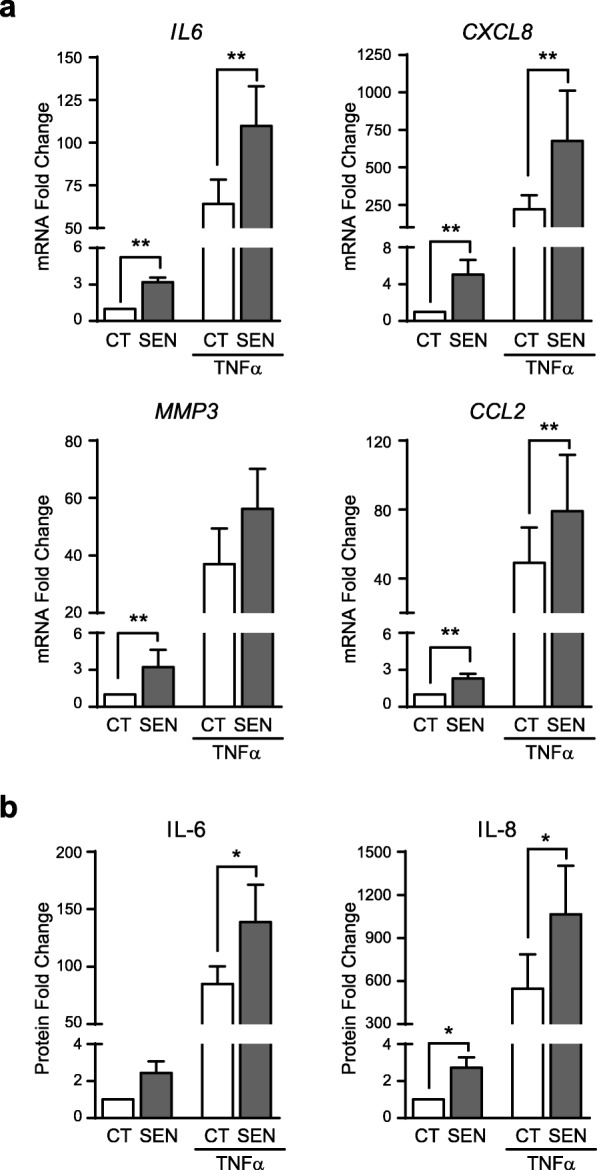
Response to an acute inflammatory damage of TNFα-induced senescent SF. 14-day senescent (SEN) and control (CT) SF were treated with TNFα. Untreated CT was used as reference. **a** Change in *IL6*, *CXCL8*, *MMP3* and *CCL2* mRNA expression (*n* = 9). **b** Change in IL-6 and IL-8 protein levels (*n* = 6). (*Wilcoxon test)

To model the potential impact of senescence on the response of SF to the inflammatory environment of arthritic joints, TNFα-induced senescent SF were stimulated with a secondary challenge of TNFα for 24 h. TNFα re-stimulation induced a significant increase in the mRNA expression of the SASP components *IL6*, *CXCL8* and *CCL2* in senescent compared to control SF (Fig. 5a). Likewise, secretion of the cytokines IL-6 and IL-8 was enhanced in senescent SF after TNFα treatment (Fig. 5b).

Collectively, these findings indicate that the response of senescent SF to further inflammatory challenge is heightened, so that senescence potentiates the expression and production of inflammatory mediators.

## Discussion

Tissue accumulation of senescent cells has been identified as a deleterious factor that promotes inflammation and tissue damage in different human diseases and animal models of aging related diseases. Regarding joint diseases, evidence of this concept has been only provided in human and experimental OA, with the main focus on chondrocytes and cartilage damage. Human cartilage and chondrocyte cultures from OA patients have shown increased number of senescent cells that contribute to cartilage degradation by increased IL-1, IL-6 and MMP-3 expression [12, 14].

We have studied cell senescence in variably aged normal ST and found an increased proportion of p16(+) cells in aged donors in the absence of inflammatory pathology, and at levels that parallel those observed in similarly aged OA or RA tissues. Furthermore, cultured normal SF exhibit levels of senescence that correlate well with the age of the donors and that are also significantly increased in the > 40 year-old group. Similar observations have previously been described in mouse and human skin fibroblasts, where senescence was associated to defective repair and a pro-inflammatory SASP, but its implications in skin disease have not been studied [31–33]. Lung fibrosis fibroblasts also show senescent features and a profibrotic and SASP phenotype that contributes to experimental lung fibrosis [22]. The contribution of senescence of other cell lineages such as macrophages, chondrocytes or cardiomyocytes, to different aging pathologies such as arteriosclerosis, OA or cardiomyopathy has also been demonstrated by senolytic intervention in animal models [15, 34, 35]. Although typical SASP cytokines and other pro-inflammatory factors are commonly represented in different cell types, cell and tissue-specific differences occur that may explain the different pathological consequences of senescence such as defective repair, fibrosis or inflammatory damage [36, 37].

Our observations suggest that synovial senescent SF accumulation occurs progressively along normal aging, from relatively early ages, and preceding the age of peak prevalence of RA. Interestingly, in old OA or RA diseased synovium no further increase was observed in the proportion of p16 senescent cells or in ex vivo cultured senescent SF compared to age-matched normal synovial tissues. Only in RA synovium from young (< 40 years old) individuals, an increased proportion of senescent cells was observed. A potential limitation of our study is that the number of control biopsies was relatively low due to their origin, i. e., healthy donors, but the sample size allowed statistical comparisons that showed significant changes. Another limitation is the limited availability of RA tissues from patients under 40 years-old. Only histological samples but not SF cultures from patients under this age were available.

We have also confirmed the capacity of oxidative stress and TNFα to induce SF senescence ex vivo. Therefore, SF senescence may be a consequence of inflammatory cell damage in younger RA patients but could also be interpreted as premature synovial cell aging preceding and contributing to RA development. The observed senescent SF phenotype includes enhanced expression of typical SASP factors with relevant roles in the pathogenesis of RA such as IL-6, IL-8, MCP-1 and MMP-3, as well as an enhanced response to further TNFα stimulation. In endothelial cells, a similar positive feedback mechanism has been described by which TNFα-mediated senescence activates the secretion of factors that sustain senescence and cytokine production via JAK/STAT pathway activation [38].

To further explore the potential of senolytic intervention to reduce the expression of pro-inflammatory factors in SF, we treated senescent SF cultures with fenofibrate, an agent that we found able to reduce cell senescence in chondrocytes. Fenofibrate reduced the expression of the senescence marker *CDKN2A* in SF cultures, confirming previous findings in tumour cell lines [29], although the mechanism is still unclear since we did not observe increased death in fenofibrate-treated senescent SF. Such reduction of *CDKN2A* expression was associated to a reduction of pro-inflammatory factors. Further studies are needed to confirm the relevance of this process in the development and progression of RA and to develop senescence based therapies.

Another process, mechanistically linked to senescence and inflammation, is the activation of a reparative program by reprograming cells with stem pluripotent capacity. This has been explored in animal models by the expression of the NANOG pluripotency marker. Mosteiro et al have elegantly described the link between senescence and reprogramming, and proposed that the senescent cells send signals to neighboring cells to promote reprogramming, with IL-6 being of particular relevance [19]. Multilineage-differentiating NANOG(+) cells have been isolated from human synovial tissues [39, 40].

Interestingly, we observed an increase of NANOG expression in inflammatory OA and RA tissues compared to control ST providing evidence of the link between this factor and the inflammatory process. However, in normal older tissues, in spite of the accumulation of senescent cells, NANOG expression was not increased which suggests that higher inflammatory, i.e. IL-6, levels are required to activate reprogramming and regeneration.

In summary, our data describe the kinetics of senescent SF accumulation in synovial tissue along normal aging and in inflammatory joint disease, and confirm the potential of these cells to contribute to the RA pathology by producing inflammatory mediators.

## Conclusions

This study provides evidence showing that senescent cells accumulate in synovial tissues in normal aging and prematurely in RA. We demonstrate that senescent SF show enhanced pro-inflammatory and tissue-damaging mediators that may contribute to the pathogenesis of RA. Therefore, removal of senescent cells or senolytic strategies could be a potential intervention to prevent or reduce synovial inflammation in RA. This adds RA and possibly other chronic arthritides to the group of diseases associated to increased tissue cell senescence where senolytic/senomorphic pharmacological interventions should be further investigated.

## Methods

### Patients and synovial biopsies

ST were obtained by arthroscopic knee biopsies from patients who fulfilled the American Rheumatism Association revised criteria for RA (*n* = 43) [41]. All patients had active disease characterized by inflammation of at least one knee joint. OA synovial tissues were obtained by synovectomy at prosthetic join replacement surgery (*n* = 18) and histologically normal synovial tissues from healthy individuals without joint disease at elective arthroscopy for minor traumatic lesions, excluding those tissues with significant inflammatory cell infiltration (*n* = 15). All patients signed a written informed consent, and the study was approved by ethics committees of Hospital Clinic, Barcelona, and Hospital 12 de Octubre, Madrid (N° CEI:17/085).

### Immunolabelling of synovial tissues

Immunohistochemical staining was performed using a standard indirect avidin-biotin peroxidase method using EnVision System-HRP Labelled Polymer (DAKO, Carpinteria, CA, USA). The following antibodies and matched isotype controls were used: anti-CDKN2A/p16INK4a (EPR1473 IgG clone, Abcam, Cambridge, UK) and anti-NANOG mAb (D73G4 IgG clone, Cell Signaling). Pretreatment included microwave heating for 15 min in Tris EDTA Tween 20 pH 9. One representative section for each synovial tissue was photographed and digitalized using a Zeiss Axiocam ERc5s camera and Zen 2012 software (Zeiss, Jena, Germany) on a Zeiss Axio Scope.A1 microscope and the number of p16 and NANOG positive cells per synovium area (mm^2^) was quantified.

Double hsp47 (anti-hsp47 mAb M16.10A1 IgG_2b_ clone, Assay Designs, Ann Arbor, MI, USA) or CD68 (anti-CD68 mAb KP1 IgG_1_ clone, DAKO) and p16 labelling by IF was also performed using sequential incubation with anti-goat IgG Alexa-Fluor 594 (red) and anti-mouse IgG_1_ Alexa-Fluor 488 (green) secondary antibodies (Invitrogen Molecular Probes, Eugene, OR, USA) respectively.

Sections were counterstained with haematoxylin or 4′,6-diamidino-2-phenylindole (DAPI) as indicated.

### SF cultures

SF cultures were established by explant growth of small biopsy fragments in Dulbecco’s modified Eagle’s medium (DMEM) supplemented with 10% heat inactivated fetal bovine serum (FBS) (Lonza, Verviers, Belgium) and used after 3rd passage.

For a model of stress-induced senescence, SF (5000 cells/cm^2^) were cultured for a total of 14 days and treated with a short course of oxidative stress with H_2_O_2_ (200 μM) (Sigma-Aldrich, Madrid, Spain) for 1 h on day 5, or exposure to TNFα (100 ng/ml) (PreproTech, Rocky Hill, NJ, USA) for 72 h on day 2. Medium was changed every other day and, on day 13, it was replaced by 0.5% FBS. RNA and culture supernatants were collected to analyze senescence and SASP mediators. Aditionally, 14-days-cultured SF were treated with fenobribrate (25 μM, 72 h) (Secalip™, Lacer SA, Spain) or TNFα (20 ng/ml, 24 h).

### Cell cycle analysis

SF (*n* = 3) were seeded in a density of 5000 cells/cm^2^ and grown for 14 days. After trypsinization SF were fixed with cold ethanol 70% and stained with propidium iodide (20 pg/ml) in presence of RNase A (Roche, Brandford, USA) 100 μg/ml at 37 °C for 30 min [42]. Finally, SF were analyzed by flow cytometry on a BD FACSCalibur instrument (Becton Dickinson, San José, CA, USA).

### Senescence-associated beta-galactosidase (SA-β-gal) assay

Quantification of senescence SF in cultures was determined by SA-β-gal assay. The assay was performed as described Debacq-Chainiaux et al [25]. Cells nuclei were counterstained with DAPI. Three to five random fields for each condition were photographed and digitalized by a Nikon Eclipse TE 2000-S microscope using the 100x objective. The total number of SF in the cultures was quantified by DAPI(+) nucleus using ImageJ software (http://rsb.info.nih.gov/ij), and percentage of senescent SA-β-gal(+) SF was determined.

### Quantitative real time-PCR (qRT-PCR) analyses

Total RNA was extracted using TRI Reagent (Invitrogen) according to the manufacturer’s protocol. For the quantification of mRNA, 1μg was used for first strand cDNA synthesis with High Capacity cDNA Transcription Kit (Applied Biosystems, Foster City, CA, USA).

For PCR amplification we used cDNA and primers added to Power Sybr Green PCR Master Mix (Applied Biosystems). The sequences of PCR primers used are listed in additional table (see Additional file 3: Table S1). β*-ACTIN* was used as an endogenous reference. qRT-PCRs were performed on an Applied Biosystems 7500 Fast Real-Time PCR System (Applied Biosystems). For relative quantification we compared the amount of target normalized to the endogenous reference using 2^-∆∆Ct^ formula (Ct = threshold cycle).

### Enzyme-linked immunosorbent assay (ELISA)

Measurement of IL-6 and IL-8 in culture supernatants were determined by single specific ELISA (Biolegend Inc., San Diego, CA, USA) according to the manufacturer protocols. The read-out for all ELISAs was carried out with a MultiskanEX plate reader (ThermoScientific).

### Statistical analysis

Data were analyzed using GraphPad Prism software v6.0 (GraphPad Software, San Diego, CA, USA). Correlation was analysed by Pearson’s test. Means were compared by one-way ANOVA with Holm-Sidak’s post hoc test, Friedman test with Dunn’s multiple comparisons, Mann-Whitney test or Wilcoxon test as appropriate. A *p* value < 0.05 was considered statistically significant (*p < 0.05, **p < 0.01, ***p < 0.001).

## Supplementary information

**Additional file 1: Figure S1.** Double hsp47/p16 and CD68/p16 labelling of synovial tissues. Representative images of synovial tissues labelled for hsp47 and CD68 (red), p16 (green) and merge. Arrows indicate senescent double positive cells for each label.

**Additional file 2: Figure S2.** Kinetics of *IL6* and *CXCL8* mRNA expression in senescent SF. SF were culture for 14 days and subjected to TNFα-induced senescence. Graphics show *IL6* and *CXCL8* mRNA levels in senescent (SEN) and control (CT) SF at the indicated time-points (*n* = 2).

**Additional file 3: Table S1.** Primer sequences used for quantitative real-time PCR analysis.

## Data Availability

Data generated or analyzed during this study are included in this published article and its supplementary information files. Data not shown are available from the corresponding author on reasonable request.

## Abbreviations

DAPI: 4′,6-diamidino-2-phenylindole
DMEM: Dulbecco’s modified Eagle’s medium
ELISA: Enzyme-linked immunosorbent assay
FBS: Fetal bovine serum
IF: Immunofluorescence
IHC: Immunohistochemistry
LDH: Lactate dehydrogenase
OA: Osteoarthritis
p16: p16INK4a
qRT-PCR: quantitative real time-PCR
RA: Rheumatoid arthritis
SASP: Senescence-associated secretory phenotype
SF: Synovial fibroblasts
ST: Synovial tissues
