# Supplementary material for: Senescent synovial fibroblasts accumulate prematurely in rheumatoid arthritis tissues and display an enhanced inflammatory phenotype
Source: Immun Ageing. 2019 Nov 5;16:29. doi: 10.1186/s12979-019-0169-4 (PMC6833299; doi:10.1186/s12979-019-0169-4)
Supplement: Supplementary file 1 — Additional file 1: Figure S1. Double hsp47/p16 and CD68/p16 labelling of synovial tissues. Representative images of synovial tissues labelled for hsp47 and CD68 (red), p16 (green) and merge. Arrows indicate senescent double positive cells for each label. [file 12979_2019_169_MOESM1_ESM.pdf]

Additional file 1:

Fig. S1.

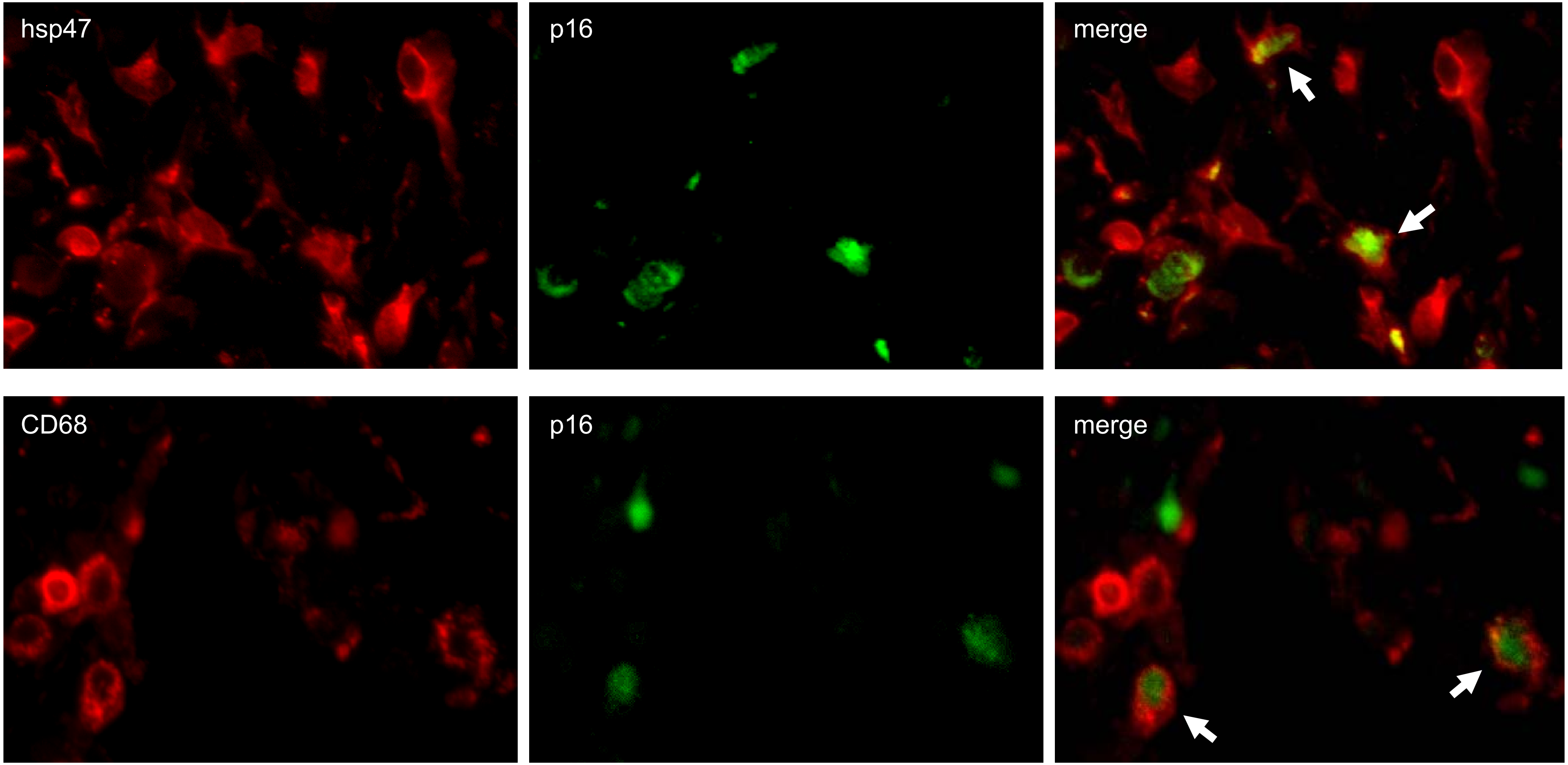

Fig.S1. Double hsp47/p16 and CD68/p16 labelling of synovial tissues. Representative images of synovial tissues labelled for hsp47 and CD68 (red), p16 (green) and merge. Arrows indicate senescent double positive cells for each label.
