# Supplementary material for: Senescent synovial fibroblasts accumulate prematurely in rheumatoid arthritis tissues and display an enhanced inflammatory phenotype
Source: Immun Ageing. 2019 Nov 5;16:29. doi: 10.1186/s12979-019-0169-4 (PMC6833299; doi:10.1186/s12979-019-0169-4)
Supplement: Supplementary file 2 — Additional file 2: Figure S2. Kinetics of IL6 and CXCL8 mRNA expression in senescent SF. SF were culture for 14 days and subjected to TNFα-induced senescence. Graphics show IL6 and CXCL8 mRNA levels in senescent (SEN) and control (CT) SF at the indicated time-points (n = 2). [file 12979_2019_169_MOESM2_ESM.pdf]

Additional file 2:

Fig. S2

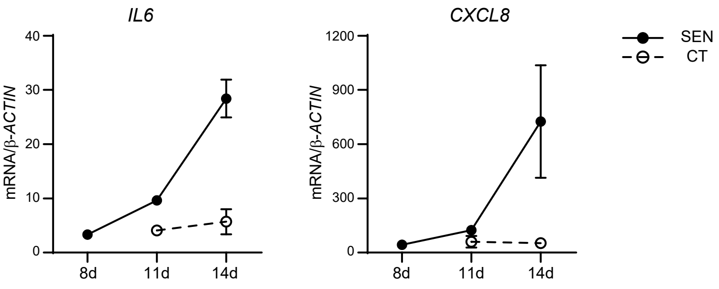

Fig. S2. Kinetics of *IL6* and *CXCL8* mRNA expression in senescent SF.
SF were culture for 14 days and subjected to TNFα-induced senescence. Graphics show *IL6* and *CXCL8* mRNA levels in senescent (SEN) and control (CT) SF at the indicated time-points (n=2).
