# Supplementary material for: Senescent synovial fibroblasts accumulate prematurely in rheumatoid arthritis tissues and display an enhanced inflammatory phenotype
Source: Immun Ageing. 2019 Nov 5;16:29. doi: 10.1186/s12979-019-0169-4 (PMC6833299; doi:10.1186/s12979-019-0169-4)
Supplement: Supplementary file 3 — Additional file 3: Table S1. Primer sequences used for quantitative real-time PCR analysis. [file 12979_2019_169_MOESM3_ESM.pdf]

Additional file 3:

Table S1. Primer sequences used for quantitative real-time PCR analysis

Gene Name	Forward Primer Sequence	Reverse Primer Sequence
IL6	5'- GTGGCTGCAGGACATGACAA -3'	5'- TGAGGTGCCCATGCTACATTT -3'
CXCL8	5’- AAGAGCCAGGAAGAAACCACC -3’	5’- CTGCAGAAATCAGGAAGGCTG -3’
MMP3	5'- CTGCTGTTGAGAAAGCTCTG -3'	5'- AATTGGTCCCTGTTGTATCCT -3'
CCL2	5’- ACTCTCGCCTCCAGCATGAA -3’	5’- TTGATTGCATCTGGCTGAGC -3’
CDKN2A	5'- GAAGGTCCCTCAGACATCCCC -3'	5'- CCCTGTAGGACCTTCGGTGAC -3'
CDKN1A	5'- GACTCTCAGGGTCGAAAACGG -3'	5'- CTTCCTCTTGGAGAAGATCAGCC -3'
LMNB1	5'- ATCGAGCTGGGCAAGT -3'	5'- TCTCGAAGCTTGATCTGG -3'
β-ACTIN	5'-GCGCGGCTACAGCTTCAC-3'	5'-GGCCATCTCTTGCTCGAAGT-3'

IL6, interleukin 6; *CXCL8*, interleukin 8; *MMP3*, matrix metallopeptidase 3; *CCL2*, monocyte chemoattractant protein 1; *CDKN2A* cyclin-dependent kinase inhibitor 2A; *CDKN1A (p21)*, cyclin-dependent kinase inhibitor 1; *LMNB1*, lamin B1
